# Simple downstream process based on detergent treatment improves yield and *in vivo* transduction efficacy of adeno-associated virus vectors

**DOI:** 10.1038/mtm.2015.24

**Published:** 2015-07-15

**Authors:** Gabriella Dias Florencio, Guillaume Precigout, Cyriaque Beley, Pierre-Olivier Buclez, Luis Garcia, Rachid Benchaouir

**Affiliations:** 1 U1179 UVSQ-INSERM, Université de Versailles Saint-Quentin en Yvelines, UFR des Sciences de la Santé, Equipe Biothérapies des Maladies Neuromusculaires, Montigny-le-Bretonneux, France

## Abstract

Recombinant adeno-associated viruses (rAAV) are promising candidates for gene therapy approaches. The last two decades were particularly fruitful in terms of processes applied in the production and purification of this type of gene transfer vectors. This rapid technological evolution led to better yields and higher levels of vector purity. Recently, some reports showed that rAAV produced by transient tri-transfection method in adherent human embryonic kidney 293 cells can be harvested directly from supernatant, leading to easier and faster purification compared to classical virus extraction from cell pellets. Here, we compare these approaches with new vector recovery method using small quantity of detergent at the initial clarification step to treat the whole transfected cell culture. Coupled with tangential flow filtration and iodixanol-based isopycnic density gradient, this new method significantly increases rAAV yields and conserves high vector purity. Moreover, this approach leads to the reduction of the total process duration. Finally, the vectors maintain their functionality, showing unexpected higher *in vitro* and *in vivo* transduction efficacies. This new development in rAAV downstream process once more demonstrates the great capacity of these vectors to easily accommodate to large panel of methods, able to furthermore ameliorate their safety, functionality, and scalability.

## Introduction

Recombinant adeno-associated viruses (rAAV) are used as tool for gene transfer applications. Clinical trials, using rAAV as gene therapy vectors, have yielded promising results^1^ and first marketing approval, using a rAAV1 vector, was delivered by the European Union in October 2012 for the treatment of familial lipoprotein lipase deficiency.^2,3^ These vectors belong to the Parvoviridae family, nonenveloped viruses embedding a single-stranded DNA genome packaged into a 25 to 30 nanometer icosahedral shaped capsid. These last two decades offered large panel of upstream and downstream methods for rAAV, aimed at constantly improving the yields, the purity, but also the scale and the time of the processes.^4^ Among them, tri-transfection remains the most popular method of rAAV production for a large number of research laboratories.^5–7^ This transient approach uses transfection of the human embryonic kidney 293 (HEK293) cell line with three different plasmids. The first harbors the transgene of interest flanked by the viral inverted terminal repeat (ITR) sequences (essential elements for genome replication and packaging), the *rep* and *cap* genes coding plasmid (for the expression of viral enzymes and structure proteins respectively) and the adenoviral helper coding plasmid (expressing the genome replication helping functions). The adherent cells can grow in many precoated tissue culture plates commercially available, offering multitude of surface areas, and giving the possibility to scale up the levels of production.^8^ Tri-transfected HEK293T cells rapidly initiate rAAV production, generally ending culture within 72 hours post-transfection, and vectors are usually recovered from cell lysate after elimination of medium.^9^ Chemical and/or physical treatments are required to release the vectors from the cells, followed by microfiltrations through specific membranes. After these clarifications steps, downstream processes can be performed by different methodologies. Among them, density gradient ultracentrifugation remains the most used method for research grade rAAV purification. Iodixanol-based isopycnic ultracentrifugation is generally preferred to the classical CsCl_2_ approach due to its higher particles recovery yields.^10,11^ Chromatographic methods, using either ion-exchange^12–14^ or affinity-based approaches are also used.^15–17^ However, unlike ultracentrifugation, chromatographic techniques are more serotype specific and need more technical adjustments to separate the full from empty particles.

It is surprising to note that, up to recently, only few studies cared to verify the amount of rAAV into the supernatant. Recent developments described the possibility to recover the viral vectors from the culture medium, avoiding the more complex and time-consuming process issue from the treatment of the cellular fraction.^18–20^ Moreover, the rAAV collected from the conditioned medium is still functional after *in vivo* delivery, despite the probable presence of proteases liberated by dead cells during production time lapse. Nevertheless, in both cases (cells versus supernatant processing), a significant amount of rAAV is inevitably lost, reducing the absolute number of vector particles to be recovered. One obvious method could be to treat both cell pellet and supernatant in parallel and pool batches after purification. However, this method will considerably increase the time of processing and eliminate the benefice brought by the simplest and faster supernatant purification method.

Here we show that the whole culture, including cells and supernatant, can be entirely processed using a very simple detergent treatment during the initial clarification step. This new method leads to higher rAAV recovery without diminishing the simplicity and fastness of the purification process. A first concentration step is performed by using a simple tangential flow filtration method, and is followed by an isopycnic density gradient. The entire process leads to high vector recovery and purity, and allows notable reduction of process duration. Moreover, the rAAV conserve efficient *in vitro* and *in vivo* functionality that surprisingly appears better than vectors purified by classical methods. This new technological approach could be easily implemented for higher production scales performed in large cell factory systems, and advantageously bridges research and pilot development activities on rAAV gene therapy vectors.

## Results

### Nonionic detergent action highly simplifies rAAV purification process

**Figure 1:**
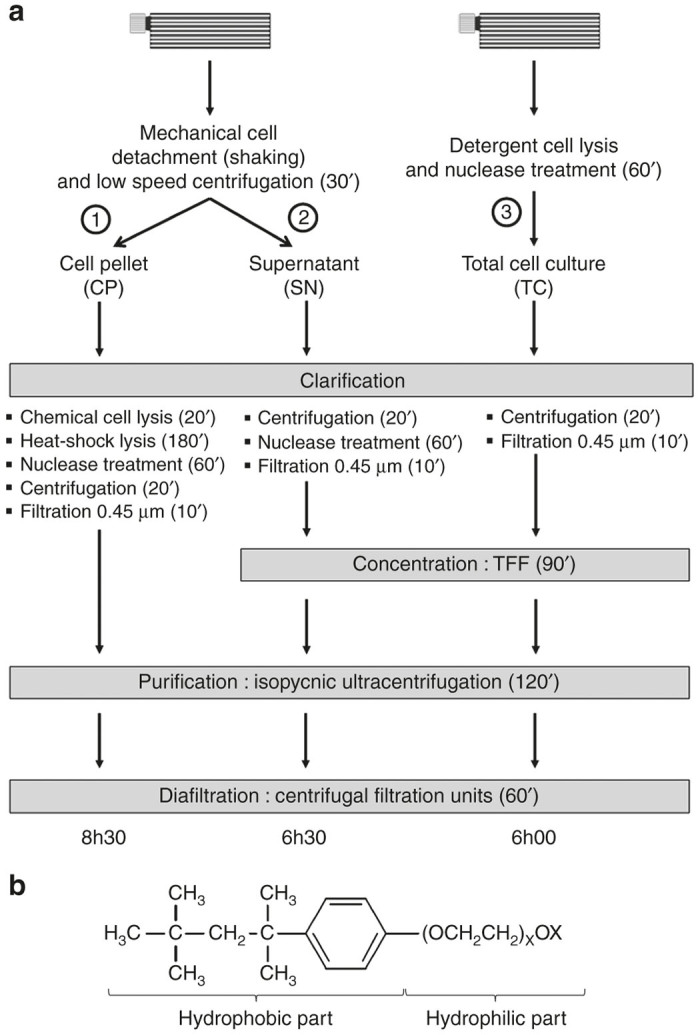
Comparison of the three different purification processes of recombinant adeno-associated virus (rAAV). (**a**) rAAVs are produced in 10-layer Hyperflask vessels by using tri-transfection method in HEK293T adherent cells. In (1) and (2), cell pellet (CP) or supernatant (SN) are separated from the same Hyperflask. Adherent cells are first detached by manual flask shaking and the cell suspension is collected. The vessel is washed once with saline buffer that is pooled with the initial cell suspension. After low speed centrifugation (500 × g), to avoid cell membrane disruption, CP and SN are treated separately. In (1), cells are chemically (lysis buffer) and physically (heat-shock) lysed before enzymatic digestion of nucleic acids. Cellular debris is pelleted by high speed centrifugation (3,500 × g) and supernatant is filtrated through a 0.45 µm pore membrane. Clarified solution is deposited on iodixanol-based discontinuous density gradient and ultracentrifuged at 500,000 × g. Recovered fractions are diafiltrated and concentrated through 100 kDa molecular weight cutoff (MWCO) centrifugal filtration units. In parallel, SN (2) is first clarified by high speed centrifugation (3,500 × g) to remove cell debris. After nucleic acids digestion, a 0.45 µm filtration is performed as final clarification step. Resulting filtrate is concentrated by tangential flow filtration (TFF) using a 500 kDa MWCO hollow fiber. Concentrate is then purified and diafiltrated as described for process (1). Our new method (3) performs cell lysis by simply adding Triton X-100 detergent and nuclease directly into the Hyperflask full of its original conditioned medium. The total cell culture (TC) is collected and the vessel is washed with saline buffer. Washing solution is pooled with the initial cell suspension and centrifuged at high velocity (3,500 × g) to remove cell debris, followed by a filtration through a 0.45 µm pore membrane. The concentration, purification, and diafiltration steps are identical to those described in method (2). Each of the three purification methods differs by the process duration, and the third avoids the loss of viral particles by recovering the totality of cell culture fractions including CP and SN. (**b**) Structural formula of Triton X-100. This nonionic chemical detergent possesses one short hydrophobic part and one hydrophilic part consisting of a series of ethylene oxide molecules (X ~ 9 to 10). See text for more technical details.

Original rAAV purification process from tri-transfected HEK293T cells has recently evolved by using supernatant rather than cell pellet to recover viral particles.^20^ The authors concluded that purification from the medium drastically diminishes the process duration compared to classical AAV harvesting from the cell pellet. Nevertheless, the sidelining of vectors encased into the cells seemed to us disadvantageous in term of yields and particle recoveries. We thus proposed an efficient alternative process based on vector recovery from the whole culture, summing particles from both cells and supernatant. As described in Figure 1a, we compared three rAAV downstream processes issued from tri-transfection of HEK293T cells cultivated in Hyperflask vessels. In each independent experiment (*n* = 4), two Hyperflasks were transfected from same transfection mixture to avoid any experimental bias due to plasmid quality or quantity. 560 ml of medium was used to completely fill one Hyperflask. The first vessel was used to recover rAAV from cell pellet (CP) after mechanical cell detachment (manual tapping and shaking) followed by a low-speed centrifugation (500 × g) to avoid physical cell lysis. The resulting supernatant (SN) was treated independently, while the second vessel was used to recover vectors from total cell culture (TC) after simple addition of Triton X-100 at 0.5% final concentration. Detergent and endonuclease (RNAse) treatments were performed at 37 °C for 1 hour under vigorous automatic Hyperflask shaking. One hour in presence of detergent was enough to completely lyse cells as determined by cell viability counting (not shown). In both cases, the original Hyperflask was washed once with 440 ml of phosphate buffered saline (PBS), meaning that 1 l of conditioned medium (SN or TC) was collected from this initial process. The clarification step showed clear difference in process duration with 5.5, 2, and 1.5 hours for CP, SN, and TC methods, respectively. Beyond this crucial parameter, it is important to note that the TC approach proposes a more ergonomic activity since it uses fewer stages before the 0.45 µm filtration process that ends all the clarification steps. In particular, the time consuming and complex heat-shock lysis method (3 hours), which comprises four sequential freeze-thaw cycles (−80 °C/37 °C), was suppressed from the SN and TC methods. Despite the concentration step performed for the SN and TC methods (Figure 1a), total process duration is significantly decreased compared to the CP method since only 6 hours are needed with TC approach to obtain highly purified vectors from one Hyperflask against 6.5 and 8.5 hours for SN and CP conditions respectively. This means that 1 day of downstream process is enough to recover highly purified AAV product. Beyond the aim for process simplification, we also pay special attention to the cost generated by our new experimental procedure. Indeed, we decided to omit the addition of DNase during the TC and SN clarification stages especially due to its prohibitive price. Surprisingly, this omission does not affect the purification process since we observed that the viscosity generated during concentration steps (tangential flow filtration (TFF)) was drastically reduced by simply adding RNAse (data not shown). Moreover, the use of an isopycnic density gradient at the end of the process, acting as a barrier for DNA sedimentation, allows removing of most of the soluble DNA in upper part of the gradient which becomes well separated from the bottomed collected fractions.^21^

### TC method significantly increases the yields without altering final purity of rAAV preparations

**Figure 2:**
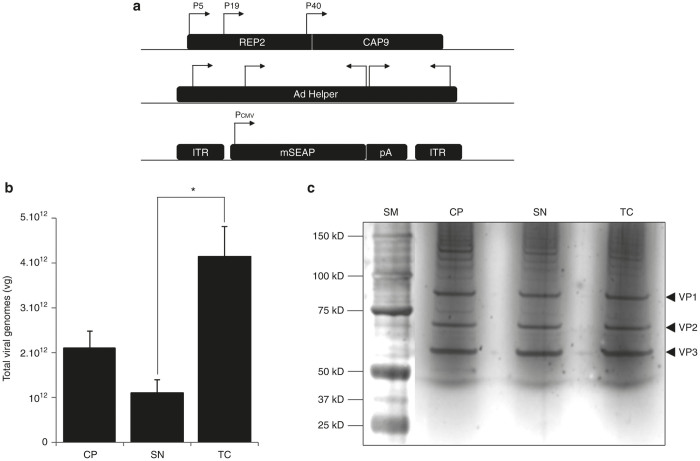
Comparison of vector yields and purity between CP, SN, and TC processes. (**a**) Schematic representation of plasmid constructions used for recombinant adeno-associated virus (rAAV) production. The pAAV2/9 plasmid (upper part) encodes for the rep and cap genes. Regulatory and structural AAV proteins are controlled by their natural P5 (Rep78/68), P19 (Rep52/40) and P40 (VP1, 2, and 3) promoters. The cap genes encode for serotype 9 capsid. The helper adenoviral plasmid (middle part) encodes for helper functions. The pAAV-mSEAP cis-plasmid (lower part) encodes for the mSEAP reporter gene under the control of CMV promoter (Pcmv) and terminated by a non constitutive polyadenylation site (pA). Transgene construction is cloned between two inverted terminal repeats (ITR) originating from AAV2 serotype. See Materials and Methods for more details about transfection protocol. (**b**) Total viral genomes (vg) recovered at the end of each type of process (CP, SN, and TC). Quantification revealed that higher rAAV recovery is achieved for TC-derived vectors, with a twofold difference between TC and CP processes (*P* = 0.134) and fourfold significant difference (*) between TC and SN process (*P* = 0.037). Refer to Materials and Methods for quantification protocol and methods of statistical analyses. Refer also to Supplementary Table S1 for titration details. (**c**) AAV vector quality determined by silver staining of SDS-PAGE. 10^10^ vg of rAAV from CP, SN, and TC processes are denatured, reduced, and migrated in 4%/12% SDS-PAGE. Silver staining of the gel reveals the three AAV capsid components materialized by 82, 67, and 60 kD bands, corresponding to VP1, VP2, and VP3 proteins respectively. Note that no other contaminating band can be distinguished on either side of the AAV capsid proteins. SM, size marker for molecular weight determination.

To evaluate the effect of detergent treatment on AAV particles, we first performed a production of rAAV2/9-mSEAP vectors by using the tri-transfection protocol as described above and also in material and methods (Figure 2a). From four independent productions, we used quantitative polymerase chain reaction (Q-PCR) to titer viral genomes (vg) from CP, SN, and TC methods. We show that number of vg was significantly higher in TC-derived vectors than from CP or SN processes (Figure 2b and Supplementary Table S1). Quality of the final AAV product was also evaluated by silver staining of 10^10^ vg issued from the three distinct processes which were deposited and migrated in parallel through sodium dodecyl sulfate 4/12% polyacrylamide gel electrophoresis (SDS-PAGE). As shown in Figure 2c, no alteration of AAV capsids (VP1, VP2, and VP3) was observed in TC-derived vectors compared to the other methods (CP and SN). Moreover, capsids ratio was respected (about 1/1/10 for VP1/VP2/VP3 respectively). We conclude that the Triton X-100 treatment allows better rAAV recovery without altering the physical quality of viral vector particles.

### TC method does not alter the *in vitro* transduction capacity of rAAV vectors

**Figure 3:**
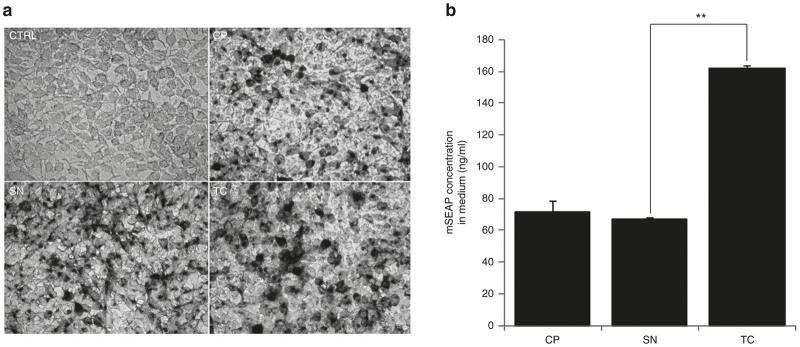
Comparison of *in vitro* transduction efficiency of recombinant adeno-associated virus (rAAV) recovered from CP, SN, or TC processes. (**a**) Light microscopy pictures of mSEAP-positive HEK293T cells 48 hours post-transduction. Compared to non transduced cells (top left), rAAV originating from TC process (bottom right) are able to efficiently transduce permissive cells and express the reporter gene as well as viral vectors recovered from the classical CP or SN processes (top right and bottom left respectively). Black points correspond to mSEAP activity revelation into transduced cells (see Materials and Methods for staining protocol). Scale bar = 40 µm. (**b**) Quantification of mSEAP activity in supernatant harvested from wells of transduced HEK293T cells. Despite the relative 2.5-fold increase of mSEAP concentration between TC and SN rAAV transduction results, this difference is still significant (with *P* < 0.01). Refer to Supplementary Table S2 for more details.

To better evaluate a prospective effect of detergent treatment in AAV functionality, we performed an *in vitro* assay by transducing permissive HEK293T cells with an equal quantity of rAAV2/9-mSEAP vectors from CP, SN, or TC processes. Because of the known low *in vitro* transduction efficiency of rAAV, we used a multiplicity of infection equal to 10^5^. Fourty-eight hours post-transduction, supernatants were collected and adherent cells were fixed for mSEAP activity revelation. Phase microscopy pictures reveal no general differences in mSEAP activity between CP-, SN-, or TC-derived vectors (Figure 3a). However, at lower magnification, we observed more product deposits in TC cell culture when compared to SN and at less extent when compared to CP (Supplementary Figure S1a). To verify these findings, we quantified the mSEAP activity in supernatants derived from these previously transduced cell cultures (Figure 3b). In accordance, we observed higher mSEAP activity in cultures transduced from TC derived rAAV with a highly significant difference (*P* < 0.01) between transduction from SN and TC vectors (66.88 ng/ml ± 0.85 and 162.03 ng/ml ± 1.84 respectively). These results suggest that TC method may better conserve high rAAV quality, increasing the amount of functional vectors.

### rAAV vectors issued from TC method perform a better *in vivo* transduction

**Figure 4:**
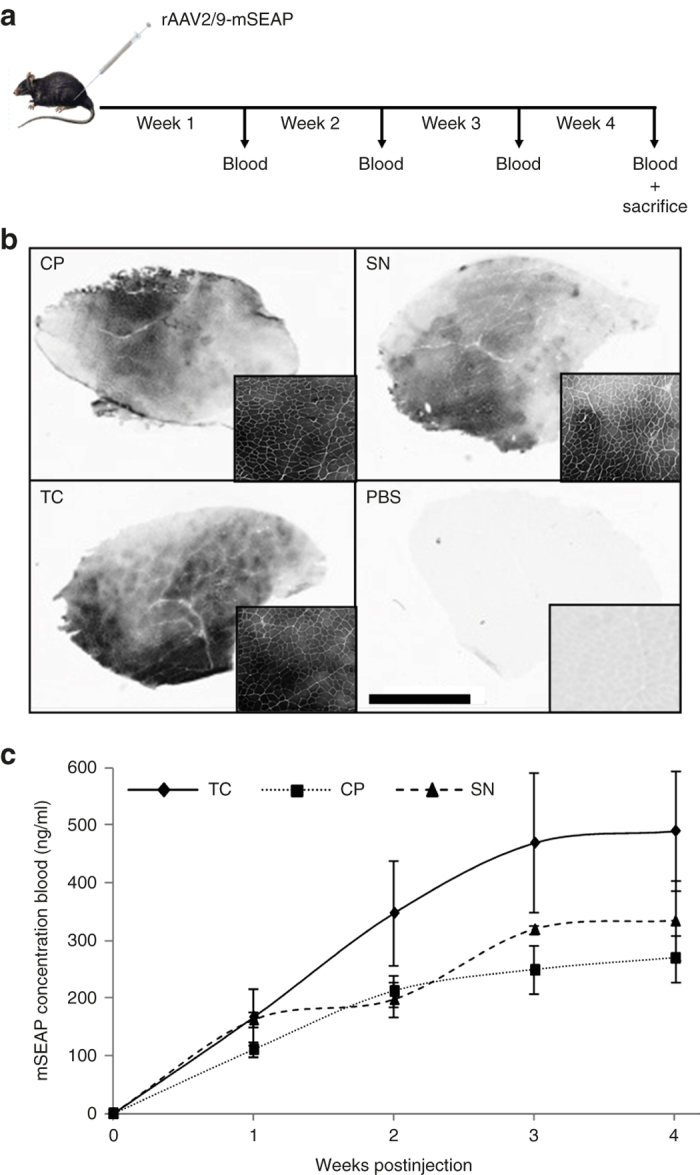
Comparison of *in vivo* transduction efficiency of recombinant adeno-associated virus (rAAV) recovered from CP, SN, or TC processes. (**a**) Schematic representation of the *in vivo* experimental protocol. C57Bl/10 adult mice are intramuscularly injected (*Tibialis anterior* (TA)) with the same suboptimal doses (10^10^ vg/TA) of CP, SN, or TC-derived rAAV, and blood is drawn weekly for one month before animal sacrifice and TA muscles recovery. Refer to Materials and Methods for detailed *in vivo* protocol. (**b**) The mSEAP revelation on TA muscle cryosections harvested 4 weeks post-transduction. Compared to PBS-injected muscle (bottom right), TC-derived vectors offer widespread TA mSEAP staining (bottom left) at level visually comparable to that of CP or SN vectors (top left and right respectively). Larger sections views are shown at bottom right of each TA muscle cryosections. Scale bar = 2 mm. (**c**) For more quantitative monitoring, *in vivo* mSEAP concentration is evaluated in blood-derived sera. From the second week postinjection, the mSEAP serum concentration average appears systematically higher in mice injected with TC-derived rAAV than with injections performed from CP- or SN-derived vectors. A stabilization of the mSEAP concentration is observed from the third week postinjection. (**b**) and (**c**) are obtained from mice injected with suboptimal doses of rAAV. Refer to Supplementary Table S3 and Supplementary Figure S1b for more detailed table and pictures from TA muscles injected at higher rAAV doses.

Beyond the *in vitro* proof of concept, we decided to evaluate the *in vivo* functionality of our rAAV vectors. Intramuscular delivery of CP-, SN-, or TC-derived vectors was performed in C57BL/10 adult mice. Two different doses were tested in order to select non saturating mSEAP concentrations and to allow better comparison of transduction efficiency (Figure 4b and Supplementary Figure S1b). Suboptimal and optimal doses were chosen based on previous studies,^22^ and rAAV2/9-mSEAP vectors were injected in *Tibialis anterior* (TA) muscles. About 50 µl of blood was weekly withdrawn from the tail vein during 4 weeks (Figure 4a). After sacrifice, cryosections of TA were stained for mSEAP product detection. Microscopic observations show that whatever the purification process used, rAAV vectors transduced the muscle with good efficiency. Nevertheless, we observed that TC-derived vectors gave better spreading of mSEAP-positive fibers and higher staining intensity around the site of injection than CP- and SN-derived vectors (Figure 4b). To confirm the histological data, a quantitative assessment was performed by measuring the kinetics of mSEAP concentration in blood during the 4 weeks postinjection (Figure 4c and Supplementary Table S3). Surprisingly, TC-derived rAAV vectors performed better *in vivo* mSEAP secretion after intramuscular delivery than those issued from other classical methods. These *in vivo* results consolidate the *in vitro* observations and confirm that our new purification method gives more functional rAAV vectors, probably due to better conservation of the vector quality and integrity.

## Discussion

rAAV downstream process is continuously evolving. Such developments allow better yields and purity, increasing the interest of these vectors for gene therapies. Here, we show that such improvements can be obtained following fast and ergonomic purification process. Instead of focussing our procedures on producer cells (CP) or on the supernatant (SN) separately, we decided to recover the vectors from the whole cell culture by using simple detergent-mediated cell lysis at the end of the production stage. Triton X-100 is a commonly used detergent in molecular and cell biology laboratories. It is a nonionic surfactant that has a hydrophilic polyethylene oxide and an aromatic hydrophobic group (Figure 1b). Its high solubility in large panel of aqueous solutions makes it the compound of choice for permeabilization of mammalian cell membranes, even in their classical culture environment. Moreover, its nonionic property is widely used to solubilize proteins while maintaining their native structure and functionality, contrary to some denaturant ionic detergents, such as SDS. One hour of 0.5% Triton X-100 treatment is sufficient for the release of intracellular rAAV from cells and added up to vector particles already present in the supernatant (originating from exocytosis or “natural” cell lysis). Our results show that Triton X-100 treatment of TC allows higher recovery of particles if compared to CP or SN purification methods. In most cases (productions #1 and #2, Supplementary Table S1) total amount of vg issued from TC procedure is equivalent to the sum of vg from CP and SN methods. Furthermore, our previous experimental observations and RNA/DNA ratio quantifications (averaged between 6 and 10) lead us to consider RNA contamination as principal cause of supernatant viscosity. This criterion, combined with the crucial role of the isopycnic iodixanol gradient as physical barrier for genomic and plasmid DNA molecules, lead us to eliminate the cost-prohibitive DNAse from the process without altering the quality of the final product. This approach, combined with reduced process duration, highly purified product, more ergonomic and less expensive procedure, constitutes a real improvement in rAAV purification method.

Release of intracellular content following cell lysis could be detrimental for the expected final purity of our viral vectors. Nevertheless, optimization of downstream technologies in this last decade has provided numerous robust and efficient equipments and disposables able to face many hurdles on purification processes. Among them, the dead-end filtration capsules, used consecutively to cell lysis, offer wide range of products with different kind of membranes (polysulfone, polyethersulfone, glass fibers, etc.) and large panel of pore sizes or molecular weight cutoff, able to significantly increase the efficiency of the clarification step in terms of debris removal and viscosity reduction (by nucleic acids capture). Otherwise, the concentration step is becoming a technology hub in the process since volume reduction simplifies the subsequent procedures, drastically diminishes the duration of the total process, and removes more contaminants. Nowadays, TFF systems propose also a wide range of fibers (hollow fibers or cartridges) with various membrane types and surfaces, and a variety of molecular weight cutoff. Moreover, complete integrated automatic systems for research or pilot scales are now commercially available, allowing perfect control of pressures, concentration levels, and flow rates. In our approach, TFF system remains central tool to treat volumes issued from cell culture vessels such as Hyperflasks, which generate up to 1 l of total cell culture suspension. By judiciously combining molecular weight cutoff, membrane surface and material assembly, TFF approach is currently able to reach 2- to 80-fold concentration levels. Such technological performances offer possibilities to easily implement rapid and low cost ultracentrifugation approaches into the process. These methods, especially those using iodixanol isopycnic density gradients^11^ remain advantageously simple not only to remove nucleic acids from fractions containing vectors but also to separate empty and full rAAV particles from initial bulk. In our hands, 1 l of clarified lysate is 30-fold concentrated before to be run through only two equilibrated ultracentrifuge tubes. According to these concentration capacities, the classical Ti70 angle-fixed ultracentrifuge rotor (Beckman Coulter, France), having eight-tube compartments, could for instance be used to treat up to 10 l of original crude lysate in one unique run of less than 2 hours. These technical characteristics offer the opportunity to use the most diffused purification approach for scalable processes as those employing baculovirus/insect cells systems,^23^ competing with some well-described chromatographic methods^12–14^ that require expensive equipment and more technical knowledge and practice than ultracentrifugation use.

The most interesting and surprising effect of the Triton X-100 treatment on producer cell lines is the generation of rAAV vectors showing better *in vitro* and *in vivo* efficiency than their homologous particles issue from classical purification methods (cell pellet or supernatant). Several hypotheses could explain this efficiency. The CP clarification process is somewhat drastic, especially during the cell lysis step. The physical cell disruption method, using several cycles of freezing/thawing can alter integrity of viral capsids without completely destroying the genome-full particles (that can still be quantified by quantitative PCR). In another hand, SN-derived vectors could be released in supernatant long before the end of the production phase. The progressive acidification of the culture medium during upstream process, resulting from the cell metabolism, in addition to the presence of proteases originating from producer cell lysis both provide an unfavorable environment for released rAAV particles and can lead to structural alteration of the SN-derived vectors. *In vivo* analyses (Figure 4c) were performed following intramuscular delivery of rAAV-mSEAP. Murine-secreted alkaline phosphatase was previously described as excellent reporter gene due to its nonimmunogenic property that allows noninvasive monitoring of gene transfer.^24,25^ In this study, we clearly show a better mSEAP product expression in blood of mice injected with TC-derived rAAV compared to those injected with SN- or CP-derived vectors. Following our argumentation, we can suppose that the detergent treatment avoids the use of techniques that alter the physical integrity of viral particles and thus enrich the proportion of fully functional rAAV.

These last years, continuous development of upstream and downstream processes has led to successful improvements of several quantitative and qualitative characteristics of rAAV as the yields, purity, and scalability. These ameliorations were also combined with reduction of process durations and reduction of the costs, two important criteria in industrial technologies dedicated to clinical aiming.

## Materials and Methods

### Upstream process

A multilayer Hyperflask (Corning, The Netherlands), with a total growth area of 1,720 cm^2^, is seeded following manufacturer instructions with 35 × 10^6^ HEK293T cells (corresponding to 2.0 × 10^4^ cells per cm^2^) in total volume of 560 ml of 1× Dulbecco’s modified Eagle medium supplemented with Glutamax, 4.5 g/l of D-Glucose (Life Technologies, France), 10% of fetal bovine serum (Biowest, France) and 1% of Penicillin-Streptomycin solution (Life Technologies). Three days postseeding, HEK293T cells are tri-transfected by mixing 1,125 µl of 10 mmol/l polyethylenimine (Sigma-Aldrich, France) with 265 µg of pAdDeltaF6 helper plasmid, 105 µg of pAAV2/9 packaging plasmid (both from Penn Vector Core, University of Pennsylvania, USA), and 130 µg of pAAV-mSEAP cis-plasmid, all (both polyethylenimine and plasmids) previously diluted in 10 ml of 150 mmol/l NaCl solution. Transfection is performed in Dulbecco’s modified Eagle medium supplemented with 2% fetal bovine serum and 1% Penicillin-Streptomycin solution. Transfected cells are incubated for 72 hours at 37 °C under 5% CO_2_.

### Downstream process

As described in this paper, we investigated new purification process and compared it to previously published methods (see Figure 1a). The first protocol uses mechanical cell detachment by manually shaking the Hyperflask. Cell suspension (560 ml) is collected and 440 ml of 1× PBS is used to wash the Hyperflask. The 1 l cell suspension is slowly centrifuged 30 minutes at 500×g to avoid physical cell lysis. Supernatant (SN) is cautiously harvested in new vessel, and cell pellet (CP) is pretreated by pipeting with 18 ml of lysis buffer (0.15 M NaCl, 50 mmol/l Tris HCl pH 8.5). CP prelysate is then processed through four cycles of freezing/thawing each consisting 30 minutes incubation in absolute ethanol cooled in dry ice followed by a 15-minute incubation in a 37 °C water bath. Nuclease treatment is performed by using 50 U/ml of benzonase (VWR, France) for 60 minutes at 37 °C. Clarification process is ended by centrifugation of cell lysate at 3,700×g for 20 minutes followed by filtration through a 0.45 µm polyethersulfone membrane syringe filter unit of 33 mm diameter (Millipore, France). The 1 l SN is treated 2 hours with 5 µg/ml of RNase A (Sigma-Aldrich, France) at 37 °C under strong magnetic shaking. SN is then filtered through a 0.8–0.45 µm sartoscale 13 cm^2^ double layer polyethersulfone membrane filter (Sartorius-Stedim, Germany).

The third protocol we investigated here drastically simplifies previous described methods. The 560 ml supernatant of the original Hyperflask is slowly collected in a vessel and treated with 3 ml of Triton X-100 nonionic detergent (Sigma-Aldrich), corresponding to 0.5% final concentration and 5 µg/ml of RNAse A (Sigma-Aldrich). After rapid homogenization, the supernatant is transfered to the original Hyperflask and incubated for 1 hour at 37 °C under vertical rotating shaking performed at 150 rpm. TC suspension is collected in a vessel and 440 ml of 1× PBS is used to wash the Hyperflask. The washing suspension is added to the first 560 ml and the 1 l volume is centrifuged at 3,700×g for 30 minutes. The supernatant derived from TC production is then filtered through the same 0.8–0.45 µm sartoscale filter than previously described for the SN filtration.

While CP filtrate is consecutively purified by isopycnic ultracentrifugation (see description below), SN and TC filtrates (1L each) are first concentrated by TFF. The TFF was processed by using the automated Kros Flow Research IIi TFF system (Spectrum Laboratories, France) through a 115 cm^2^ polyethersulfone membrane hollow fiber unit with 500 kDa molecular weight cut off. SN and TC filtrates are concentrated 30 times, corresponding to a final volume of 35 ml.

The three methods are ended by an isopycnic ultracentrifugation derived from previously described protocol.^11^ Briefly, 17 ml of viral suspension is deposited through a glass Pasteur pipette in the bottom of 25 × 89 mm Quick-Seal Polyallomer dome-top tube (Beckman Coulter, France). This suspension is raised up by successive addition of 9 ml of 15% iodixanol (from 60% stock solution; Sigma-Aldrich) at 1M NaCl, 5 ml of 25% iodixanol, 5 ml of 40% iodixanol, and 3 ml of 60% iodixanol. Iodixanol solutions are all diluted in 1× PBS-MK buffer (1M NaCl, 1 mmol/l MgCl_2_, 2.5 mmol/l KCl, 1× PBS). Tubes are heat-sealed with the Tube topper system (Beckman Coulter), placed in Ti70 rotor, and ultracentrifuged at 500,000×g (70,000 rpm) for 70 minutes in an Optima L100XP ultracentrifuge (Beckman Coulter). The Fraction Recovery system (Beckman Coulter) is used to puncture the tube from the bottom side: the six first milliliters, containing viral particles, are collected and diluted twice in 1× PBS before their diafiltration through centrifugal concentration and filtration unit Amicon Ultra-15 (Millipore, France) according to the manufacturer instructions. The diafiltration is performed three times leading to a 450 times dilution factor of the original collected solution. This step is ended by a 6 to 10 times concentration step (0.9 to 1.8 ml final volume).

### Vector genome titration by Q-PCR

5 µl of purified vector is treated 30 minutes at 37 °C with 5 µl of DNAse I at 2U/µl (Ambion) diluted in 40 µl of DNAse buffer (13 mmol/l TRIS pH7.5, 0.12 mmol/l CaCl_2_, 5 mmol/l MgCl_2_). Five microliters of Proteinase K at 20 mg/ml (Ambion) diluted in 195 µl of Proteinase K buffer (0.5% SDS, 10 mmol/l EDTA) are added to previous mixture, followed by 1 hour incubation at 56 °C. Ten microliters of pretreated solution are serially diluted to obtain 10^–2^ to 10^–7^ of AAV DNA dilutions. Taqman Q-PCR is performed by using the iTaq Universal Probes Supermix (BioRad), 0.1 µmol/l of AAV22mers forward primer (5′-CTCCATCACTAGGGGTTCCTTG-3′), 0.3 µmol/l of AAV12mers reverse primer (5′-GTAGATAAGTAGCATGGC-3′) and 0.1 µmol/l of AAV_MGB.P Fam Taqman probe (5′-TAGTTAATGATTAACCC-3′, Life Technologies). Fifteen microliters of the previous Taqman mix are added to 10 µl of diluted AAV DNA. In parallel, 10 µl of serially diluted AAV plasmid, containing ITR2 sequences (from 10^8^ to 10^2^ copies per well) is deposited in duplicates to serve as plasmid range. PCR is run in CFX96 Touch Real-Time PCR Detection System (BioRad) under following conditions: hold at 95 °C for 15 minutes, and 40 cycles at 95 °C/15 seconds (denaturation) and 54 °C/1 minute (hybridization). Raw data were exported and analyzed by the BioRad CFX manager software. Titration results are given in viral genomes/ml (vg/ml).

### Silver staining of SDS-PAGE

Purity of recombinant AAV vectors is assessed by loading 1 × 10^10^ vg on SDS-PAGE (Life Technologies) run under reducing conditions. Proteins and nucleic acid content is revealed by using Silver Staining Plus protocol (Bio-Rad Laboratories, France) following manufacturer instructions.

### *In vitro* assay

*In vitro* assays are performed in 24-well tissue culture plates having 2 cm^2^ of growth area per well. 2 × 10^5^ HEK293T cells per well are seeded in total volume of 300 µl of complete medium (described before). After 24 hours, an average of the number of cells per well is determined by trypsinization and automatic cell counting. Vector transduction is performed at multiplicity of infection 10^5^. Fourty-eight hours later, the medium is collected and conserved at −20 °C until use. The cell monolayers are washed once with 1× PBS and fixed with 0.5% glutaraldehyde solution for 15 minutes at room temperature before three washing cycles of 15 minutes with 1× PBS. To inactivate endogenous alkaline phosphatase activity, cells are covered with 500 µl of PBS and plates are sealed and immersed in water bath heated at 65 °C for 30 minutes. After two PBS washings, 5-Bromo-4-Chloro-3 Indolyphosphate/Nitro Blue Tetrazolium solution (Sigma-Aldrich) is added in each well and plates are incubated at 37 °C for 30 minutes to several hours until occurrence of significant visual colored staining, compared to nontransduced cells. The mSEAP quantification in collected medium is performing with the Phospha-Light SEAP Reporter Gene Assay System (Life Technology) according to manufacturer instructions. Briefly, medium is first 10-fold diluted in a dilution buffer provided with the Phosphalight kit before endogenous alkaline phosphatase inactivation at 65 °C for 30 minutes. After sixfold serial dilutions in assay and reaction buffers containing chemiluminescent substrates and enhancers, microplates are placed in luminometer for luminescence detection. Raw data were analyzed through simple spreadsheet and quantified by using standard curve previously prepared by serially diluting stock enzyme.

### *In vivo* assay

All studies on mice were conducted in strict accordance with the institutional guidelines for animal research transposed from the European directive 2010/63/EU. Project authorization, including all the procedures, was submitted to Ministry of Higher Education and Research on March 31, 2014. All injections were performed after inhalational anesthesia using isoflurane, and all efforts were made to minimize animal number, procedure lengths, and suffering.

Ten-week-old C57BL/10 mice (three animals per group) were injected intramuscularly with 50 µl of AAV2/9-mSEAP vectors issued from CP, SN, or TC purification process. A total of 5.0 × 10^10^ vg (high dose) and 1.0 × 10^10^ vg (suboptimal low dose) were injected in left and right TA muscles of each mouse respectively. Fifty microliters of blood were withdrawn every week for 1 month, and serum was isolated by blood centrifugation at 10,000 × g for 5 minutes at 4 °C. Mice were sacrificed 1 month postinjection by cervical elongation and TA muscles are harvested.

TA muscles are immediately frozen in liquid nitrogen-cooled isopentan solution and cryopreserved at −80 °C. Serial 10-µm cryosections were made and examined for alkaline phosphatase enzymatic activity. Histological slides are first fixed with 0.5% glutaraldehyde solution for 15 minutes at room temperature before three cycles of 15 minutes washing with 1× PBS. To inactivate endogenous alkaline phosphatase activity, slides are incubated at 65 °C for 30 minutes in PBS, followed by two PBS washings. 5-Bromo-4-Chloro-3 Indolyphosphate/Nitro Blue Tetrazolium solution (Sigma-Aldrich) is added to cryosections and incubated at 37 °C for 30 minutes to several hours until occurrence of significant visual colored staining, compared to slides of noninjected muscles.

Quantification of mSEAP from mice serum is performed as described for culture medium. The only difference is the initial serum is fivefold more diluted than used with supernatant from *in vitro* experiments.

### Statistical analyses

Graphical representations are presented as mean ± standard error, and a two-tailed Student’s *t*-test is used to determine significant differences in total viral genome recovery between TC- and SN-derived vectors (Figure 2b) or for *in vitro* mSEAP product quantification between TC- and SN-derived vectors (Figure 3b). *P* < 0.05 (represented by * in Figure 2b) and *P* < 0.01 (represented as ** in Figure 3b) are considered as significant and highly significant respectively.

## References

[bib1] Kotterman MA Schaffer DV 2014 Engineering adeno-associated viruses for clinical gene therapy Nat Rev Genet 15 445 451 24840552 10.1038/nrg3742 PMC4393649

[bib2] Gaudet D Méthot J Déry S Brisson D Essiembre C Tremblay G 2013 Efficacy and long-term safety of alipogene tiparvovec (AAV1-LPLS447X) gene therapy for lipoprotein lipase deficiency: an open-label trial Gene Ther 20 361 369 22717743 10.1038/gt.2012.43 PMC4956470

[bib3] Salmon F Grosios K Petry H 2014 Safety profile of recombinant adeno-associated viral vectors: focus on alipogene tiparvovec (Glybera®) Expert Rev Clin Pharmacol 7 53 65 24308784 10.1586/17512433.2014.852065

[bib4] Ayuso E Mingozzi F Bosch F 2010 Production, purification and characterization of adeno-associated vectors Curr Gene Ther 10 423 436 21054248 10.2174/156652310793797685

[bib5] Xiao X Li J Samulski RJ 1998 Production of high-titer recombinant adeno-associated virus vectors in the absence of helper adenovirus J Virol 72 2224 2232 9499080 10.1128/jvi.72.3.2224-2232.1998 PMC109519

[bib6] Grimm D Kern A Rittner K Kleinschmidt JA 1998 Novel tools for production and purification of recombinant adenoassociated virus vectors Hum Gene Ther 9 2745 2760 9874273 10.1089/hum.1998.9.18-2745

[bib7] Salvetti A Orève S Chadeuf G Favre D Cherel Y Champion-Arnaud P 1998 Factors influencing recombinant adeno-associated virus production Hum Gene Ther 9 695 706 9551617 10.1089/hum.1998.9.5-695

[bib8] Liu YL Wagner K Robinson N Sabatino D Margaritis P Xiao W 2003 Optimized production of high-titer recombinant adeno-associated virus in roller bottles Biotechniques 34 184 189 12545558 10.2144/03341dd07

[bib9] Grieger JC Choi VW Samulski RJ 2006 Production and characterization of adeno-associated viral vectors Nat Protoc 1 1412 1428 17406430 10.1038/nprot.2006.207

[bib10] Hermens WT ter Brake O Dijkhuizen PA Sonnemans MA Grimm D Kleinschmidt JA 1999 Purification of recombinant adeno-associated virus by iodixanol gradient ultracentrifugation allows rapid and reproducible preparation of vector stocks for gene transfer in the nervous system Hum Gene Ther 10 1885 1891 10446928 10.1089/10430349950017563

[bib11] Zolotukhin S Byrne BJ Mason E Zolotukhin I Potter M Chesnut K 1999 Recombinant adeno-associated virus purification using novel methods improves infectious titer and yield Gene Ther 6 973 985 10455399 10.1038/sj.gt.3300938

[bib12] Urabe M Xin KQ Obara Y Nakakura T Mizukami H Kume A 2006 Removal of empty capsids from type 1 adeno-associated virus vector stocks by anion-exchange chromatography potentiates transgene expression Mol Ther 13 823 828 16473554 10.1016/j.ymthe.2005.11.024

[bib13] Qu G Bahr-Davidson J Prado J Tai A Cataniag F McDonnell J 2007 Separation of adeno-associated virus type 2 empty particles from genome containing vectors by anion-exchange column chromatography J Virol Methods 140 183 192 17196264 10.1016/j.jviromet.2006.11.019

[bib14] Okada T Nonaka-Sarukawa M Uchibori R Kinoshita K Hayashita-Kinoh H Nitahara-Kasahara Y 2009 Scalable purification of adeno-associated virus serotype 1 (AAV1) and AAV8 vectors, using dual ion-exchange adsorptive membranes Hum Gene Ther 20 1013 1021 19534598 10.1089/hum.2009.006

[bib15] Anderson R Macdonald I Corbett T Whiteway A Prentice HG 2000 A method for the preparation of highly purified adeno-associated virus using affinity column chromatography, protease digestion and solvent extraction J Virol Methods 85 23 34 10716335 10.1016/s0166-0934(99)00150-0

[bib16] Auricchio A Hildinger M O’Connor E Gao GP Wilson JM 2001 Isolation of highly infectious and pure adeno-associated virus type 2 vectors with a single-step gravity-flow column Hum Gene Ther 12 71 76 11177544 10.1089/104303401450988

[bib17] Koerber JT Jang JH Yu JH Kane RS Schaffer DV 2007 Engineering adeno-associated virus for one-step purification via immobilized metal affinity chromatography Hum Gene Ther 18 367 378 17437357 10.1089/hum.2006.139

[bib18] Lock M Alvira M Vandenberghe LH Samanta A Toelen J Debyser Z 2010 Rapid, simple, and versatile manufacturing of recombinant adeno-associated viral vectors at scale Hum Gene Ther 21 1259 1271 20497038 10.1089/hum.2010.055 PMC2957274

[bib19] Vandenberghe LH Xiao R Lock M Lin J Korn M Wilson JM 2010 Efficient serotype-dependent release of functional vector into the culture medium during adeno-associated virus manufacturing Hum Gene Ther 21 1251 1257 20649475 10.1089/hum.2010.107 PMC2957237

[bib20] Doria M Ferrara A Auricchio A 2013 AAV2/8 vectors purified from culture medium with a simple and rapid protocol transduce murine liver, muscle, and retina efficiently Hum Gene Ther Methods 24 392 398 24116943 10.1089/hgtb.2013.155 PMC3869536

[bib21] Dorin M Bornecque CA 1995 Fast separations of plasmid DNA using discontinuous gradients in the preparative ultracentrifuge Biotechniques 18 90, 92 7702860

[bib22] Le Hir M Goyenvalle A Peccate C Précigout G Davies KE Voit T 2013 AAV genome loss from dystrophic mouse muscles during AAV-U7 snRNA-mediated exon-skipping therapy Mol Ther 21 1551 1558 23752313 10.1038/mt.2013.121 PMC3734654

[bib23] Urabe M Ding C Kotin RM 2002 Insect cells as a factory to produce adeno-associated virus type 2 vectors Hum Gene Ther 13 1935 1943 12427305 10.1089/10430340260355347

[bib24] Bartoli M Poupiot J Goyenvalle A Perez N Garcia L Danos O 2006 Noninvasive monitoring of therapeutic gene transfer in animal models of muscular dystrophies Gene Ther 13 20 28 16107863 10.1038/sj.gt.3302594

[bib25] Maelandsmo GM Ross PJ Pavliv M Meulenbroek RA Evelegh C Muruve DA 2005 Use of a murine secreted alkaline phosphatase as a non-immunogenic reporter gene in mice J Gene Med 7 307 315 15515146 10.1002/jgm.666

