# Supplementary material for: Simple downstream process based on detergent treatment improves yield and in vivo transduction efficacy of adeno-associated virus vectors
Source: Mol Ther Methods Clin Dev. 2015 Jul 15;2:15024–. doi: 10.1038/mtm.2015.24 (PMC4502676; doi:10.1038/mtm.2015.24)
Supplement: Supplementary Figure and Tables [file mtm201524-s1.pdf]

Supplementary material

Production	Purification	Titration (vg / ml)	Final volume (ml)	Total vg
Prod #1	CP	$2,77.10^{12}$	0,84	$2,32.10^{12}$
	SN	$9,25.10^{11}$	0,84	$7,77.10^{11}$
	TC	$1,88.10^{12}$	1,68	$3,15.10^{12}$
Prod #2	CP	$3,98.10^{12}$	0,87	$3,46.10^{12}$
	SN	$2,41.10^{12}$	0,87	$2,09.10^{12}$
	TC	$3,81.10^{12}$	1,75	$6,66.10^{12}$
Prod #3	CP	$1,53.10^{12}$	0,75	$1,15.10^{12}$
	SN	$1,80.10^{12}$	0,75	$1,35.10^{12}$
	TC	$2,88.10^{12}$	1,5	$4,32,10^{12}$
Prod #4	CP	$1,64.10^{12}$	0,9	$1,48.10^{12}$
	SN	$2,10.10^{11}$	0,9	$1,89.10^{11}$
	TC	$1,39.10^{12}$	1,78	$2,47.10^{12}$

Table S1: titer comparison between CP, SN and TC processes (n=4). Final volume column refers to final product volume after the last diafiltration and concentration process (see Fig. 1a). Graphical view and statistical analyses are shown in Fig. 2b.

Purification	Experiment	MOI	Targeted cells	[mSEAP] (ng/ml)	Mean / Exp (ng/ml)	Total Mean (+/- SEM)
CP	#1	1,00.10^5	HEK293T	92,04<br>70,30	81,17	71,15<br>(+/- 7,08)
	#2	1,00.10^5	HEK293T	56,93<br>65,36	61,14	
SN	#1	1,00.10^5	HEK293T	74,22<br>57,12	65,67	66,88<br>(+/- 0,85)
	#2	1,00.10^5	HEK239T	63,77<br>72,39	68,08	
TC	#1	1,00.10^5	HEK293T	160,84<br>168,43	164,63	162,03<br>(+/- 1,84)
	#2	1,00.10^5	HEK293T	170,25<br>148,60	159,42	

Table S2: detailed *in vitro* results of mSEAP concentration in supernatant of HEK293T cells transduced with CP, SN or TC derived AAV vectors at a MOI of 10^5. Mean concentration is calculated from two distinct experiments performed in duplicate. Graphical view and statistical analyses are done in Fig. 3B.

		mSEAP concentration in blood (ng/ml)								
**Purification**	**Mice**	**Day 0**	**Week 1**	**Mean 1 (+/- SD)**	**Week 2**	**Mean 2 (+/- SD)**	**Week 3**	**Mean 3 (+/- SD)**	**Week 4**	**Mean 4 (+/- SD)**
**PBS**	#1	0	0	0	0	0	0	0	0	0
	#2	0	241,8		486,3		590,9		595,6	
**TC**	#3	0	142,1	167,5 (+/- 49,54)	297,8	348,2 (+/- 92)	*ND*	470,2 (+/- 120,73)	386,2	490,9 (+/- 104,67)
	#4	0	118,6		260,7		349,5		*ND*	
	#5	0	132,2		254,1		257,7		288,8	
**CP**	#6	0	95,1	111,1 (+/- 14,06)	183,2	212,4 (+/- 27,75)	185,5	249,2 (+/- 42,47)	209,7	269,7 (+/- 40,04)
	#7	0	106		200,1		304,4		310,7	
	#8	0	176,2		242,1		311,7		403,1	
**SN**	#9	0	149,1	162,6 (+/- 13,58)	175,9	198,1 (+/-29,37)	*ND*	319,6 (+/- 7,95)	265,7	334,4 (+/- 68,73)
	#10	0	*ND*		176,2		327,6		*ND*	

Table S3: detailed results of mSEAP concentration (ng/ml) in blood of intramuscularly injected mice. C57BL/10 adult mice are injected in TA at day 0 and blood is collected every week for 4 weeks. Group of 3 mice are injected with CP, SN or TC derived AAV vectors (one control mouse was injected with PBS), and mean of mSEAP concentration is calculated with standard deviation (SD). Some points were not determined (ND) due the low quantity of blood removed from the tail vein incision the day of sampling (see material and methods for more details). Graphical view and statistical analyses are described in Fig. 4c.

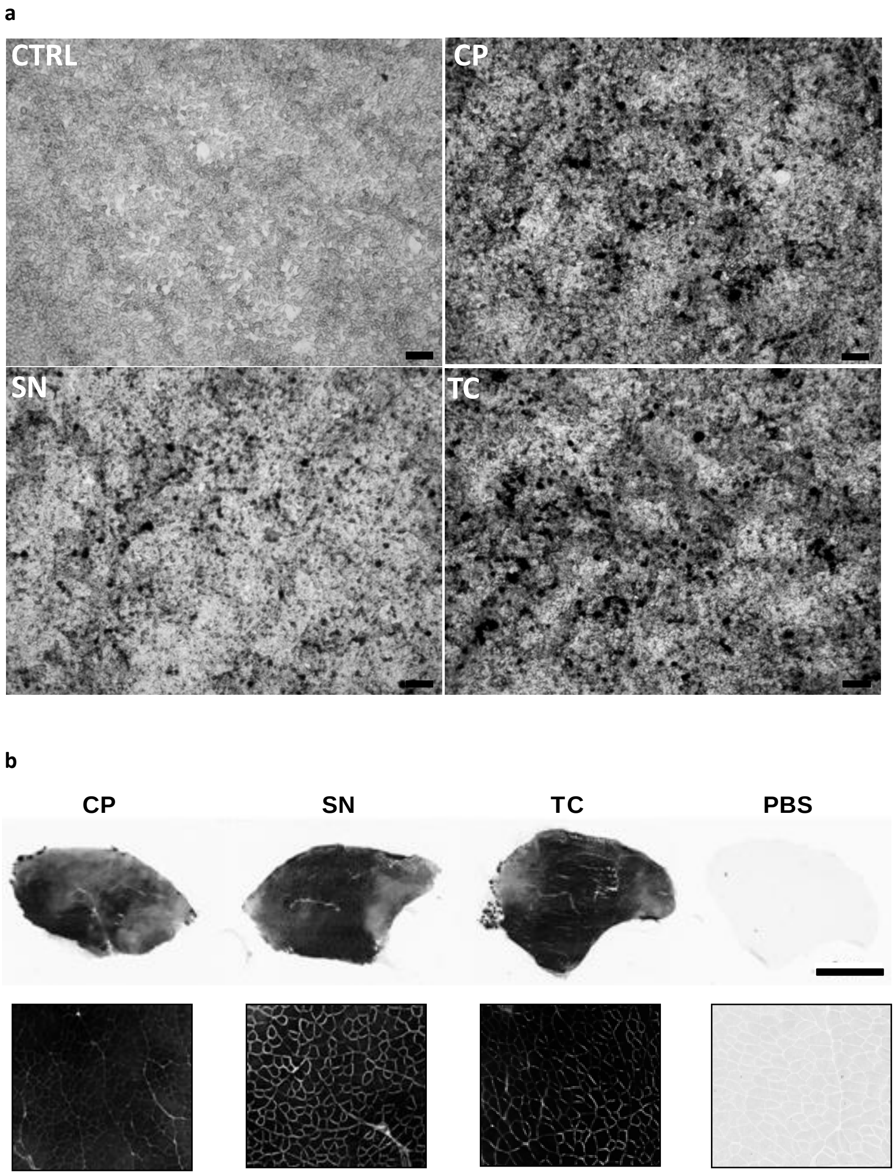

<u>Figure S1:</u> ***in vitro* and *in vivo* analyses of mSEAP activity. (a)** Light microscopic pictures of cells 48 hours post-transduction seen at lower magnification (4X objective) compared to Fig. 3a (using 20X objective). Despite relative equal number of tranduced cells (black points), higher mSEAP deposit is systematically observed in cells transduced with TC derived vectors (bottom right). Staining of non transduced cell culture is shown in left. Scale bar = 80 µm. **(b)** Light microscopy pictures of TA injected with optimal doses of CP, SN or TC derived vectors. Compared to suboptimal doses (Fig. 4b), the mSEAP revelation gives over-saturated staining, making difficult the comparison of efficiency between the three types of vectors. Scale bar = 2 mm.
